# Epigenetic Mechanisms in *Apis melifera*: From Development to Environmental Adaptation

**DOI:** 10.3390/cimb47070554

**Published:** 2025-07-17

**Authors:** Xiexin Hu, Jing Xu, Kang Wang

**Affiliations:** 1 College of Bioscience and Biotechnology, Yangzhou University, Yangzhou 225009, China; huxiexin@outlook.com (X.H.); jingxu250872@163.com (J.X.); 2 College of Animal Science and Technology, Yangzhou University, Yangzhou 225009, China

**Keywords:** epigenetics, honeybees, DNA methylation, histone modification, non-coding RNAs

## Abstract

Epigenetics, as an important scientific field that bridges genomic function and phenotypic plasticity, increasingly demonstrates its value in bee research. In recent years, with the rapid development of omics technologies, there have been significant advancements in the study of epigenetics in honeybees. This article reviews the role of epigenetic regulation in the development, behavioral regulation, and immune response of honeybee larvae from the perspectives of DNA methylation, histone modification, and non-coding RNA. With the continuous deepening of related research, honeybee epigenetics not only opens new paths for understanding the formation mechanisms of complex traits in social insects but also provides solid theoretical support and innovative perspectives for the study of social insects and beekeeping practices. These insights also inform sustainable beekeeping practices.

## 1. Introduction

The honeybee (*Apis mellifera*), as exemplified in Insights into Social Insects from the Genome of the Honeybee. The honeybee, a model social insect, exhibits a complex group structure and a significant division of labor. As a pivotal model insect, honeybees consistently attract extensive attention for their remarkable coordination of collective behavior at the colony level and pronounced phenotypic diversity at the individual level [1]. Of particular interest is the phenotypic dichotomy between queens and workers; despite both deriving from genetically identical, fertilized eggs, they exhibit profound differences in lifespan, morphology, reproductive capacity, and behavior [2]. This process is closely associated with DNA methylation, non-coding RNAs, and histone modifications [3,4,5], thereby challenging the classical genetic paradigm that posits a direct, deterministic relationship between genotype and phenotype. Epigenetics refers to the heritable regulation of gene expression that occurs without alterations to the underlying DNA sequence [6]. As a crucial regulatory mechanism linking the genome and phenotype, epigenetics is emerging as a key research direction for uncovering the plasticity of bee phenotypes and the regulatory mechanisms underlying complex social behaviors.

In recent years, the rapid development of omics technologies, such as methylome sequencing (for DNA methylation profiling) and chromatin immunoprecipitation followed by sequencing (ChIP-seq, for studying protein-DNA interactions), has enabled comprehensive insights into gene regulation and epigenetic mechanisms. This review aims to provide a comprehensive overview of the current understanding and functional characterization of the primary epigenetic mechanisms in *Apis mellifera*, including DNA methylation, non-coding RNAs, and histone modifications. It focuses on elucidating their roles in individual development, behavioral regulation, and environmental adaptation. In addition, the article summarizes the methodological limitations and future directions in this field, aiming to provide theoretical support and new insights for subsequent studies.

## 2. DNA Methylation

Since its initial discovery in bacteria in 1925, DNA methylation has become one of the earliest and most extensively studied epigenetic regulatory mechanisms. DNA methylation primarily refers to the catalyzed addition of a methyl group to cytosine, forming 5mC [7]. In most animals, this modification predominantly occurs at CpG dinucleotide sites and can influence the accessibility of promoter regions, the binding affinity of transcription factors, and the chromatin structure, thereby regulating transcriptional activity.

**Figure 1:**
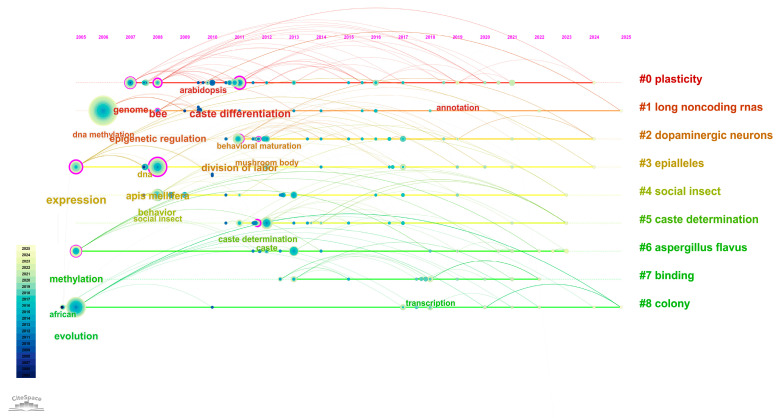
Bibliometric Analysis of Research on DNA Methylation in Honeybees. This figure presents a bibliometric mapping of research topics related to “honeybee” and “DNA methylation” based on a keyword co-occurrence analysis of publications indexed in the Web of Science database. The network illustrates the strength of associations between keywords and research themes, with node size reflecting keyword frequency and line thickness indicating co-occurrence strength. The color gradient represents the average publication year for each keyword cluster, where lighter colors denote more recent research activity. This visualization highlights evolving research trends and emerging focal points in the field of honeybee epigenetics.

DNA methylation is characterized by its reversibility, heritability, and cell-type specificity [8,9]. It plays a critical role in various biological processes including organismal development, cellular differentiation, X chromosome inactivation, regulation of imprinted genes, and tumorigenesis. In this study, we conducted a hotspot analysis based on the keywords “honeybee” and “methylation” using the Web of Science database to visualize research trends in this area (Figure 1).

### 2.1. Characteristics of DNA Methylation in Honeybees

The DNA methylation system of bees exhibits both high evolutionary conservation and unique characteristics among insects, with distinct structural and functional features. Unlike the promoter methylation patterns typically observed in mammals, DNA methylation in honeybees is enriched in gene bodies, particularly exons [10] and within exonic sequences [2,11,12], while methylation is relatively sparse in promoter and transposable element regions. This atypical distribution pattern is believed to be closely associated with the regulation of alternative splicing and the stabilization of gene expression, rather than functioning merely as a transcriptional “on–off” switch [13]. Moreover, bee DNA methylation mainly involves 5-methylcytosine [14], but its overall genomic abundance is notably low—only about 1% of cytosine residues are methylated [15,16]. This contrasts sharply with levels observed in mice (74%), zebrafish (80%), and ants (3–5%) [17].

From the perspective of DNA methyltransferase composition, the honeybee (*Apis mellifera*) exhibits a remarkably simplified yet functionally distinct epigenetic system. It retains only two principal members of the DNA methyltransferase (DNMT) family: DNMT1, responsible for maintaining methylation patterns after DNA replication, and DNMT3, which establishes de novo methylation marks [18,19]. In contrast to mammals, honeybees lack DNMT2 and other functionally redundant DNMTs commonly found in vertebrates [20,21,22]. This minimal yet well-defined enzymatic repertoire offers a tractable model for investigating the mechanisms of targeted methylation regulation and its roles in development. Functionally, DNA methylation in honeybees differs from that in other insects such as *Drosophila*, which virtually lack functional methylation systems. In honeybees, DNA methylation actively represses gene expression [23] and plays a critical role in regulating alternative splicing (AS), particularly in tissue-specific contexts [24]. For example, in worker bees, alternative splicing of the *gemini* gene—including a 9 base pair deletion—enables individuals to evade pheromone-induced ovarian suppression, thereby permitting reproductive activity [25]. This demonstrates how DNA methylation not only regulates transcription but also affects post-transcriptional processes that underline reproductive plasticity and caste-specific behaviors.

### 2.2. The Role of DNA Methylation in Caste Differentiation and Social Behavior in Honeybees

#### 2.2.1. Functional Roles

DNA methylation, as a core epigenetic mechanism underlying neural plasticity and behavioral regulation in bees, influences the expression and function of numerous cognition-related genes. Experimental studies have shown that treatment with DNA methyltransferase inhibitors, such as Zebularine, does not impair basic learning abilities in worker bees, but significantly impairs long-term memory retention consolidation [26,27]. This effect is closely associated with the specific upregulation of *Dnmt3* expression in brain regions following associative learning training. Further methylome analyses revealed methylation changes in the promoter and exonic regions of several genes implicated in memory and neural plasticity—such as *neurexin* *I*, *Dnmt3*, and *CREB*—which suggest their potential roles in the molecular regulation of memory formation [28]. At the level of behavioral plasticity, epigenomic reprogramming occurs alongside social role transitions in bees. Phenotypic reversion experiments between nurse bees and foragers identified 57 differentially methylated regions (DMRs) exhibiting reversible changes, implying that epigenetic mechanisms may serve as a form of “molecular memory” in maintaining behavioral states [29]. In response to threat stimuli, rapid methylation reprogramming occurs in the bee brain, involving genes related to synaptic plasticity and chromatin remodeling, accompanied by a significant increase in methylation entropy [30]. These dynamically methylated sites are enriched in binding domains for neurodevelopmental transcription factors such as bHLH and SOX, indicating that DNA methylation may function through constructing an epigenetic–transcription factor interaction network that finely tunes behavioral outputs in response to environmental cues. The *obp11* gene, which encodes an odorant-binding protein involved in olfactory signal transduction, is implicated in task-specific sensory perception in honeybee workers; for instance, it contains three CpG sites near the donor splice site of intron 3 that exhibit significantly higher methylation levels in female bees. This modification may influence the transcriptional process, promote the production of truncated transcripts, and modulate olfactory function through alternative splicing [31]. Similarly, CpG island regions in neuroplasticity-related genes such as *dynactin*, *nadrin*, and *pkcbp1* undergo dynamic methylation changes that alter transcription factor binding accessibility, thereby affecting behavioral expression [32]. The methylation state is also closely linked to social behavioral phenotypes. For example, methylation levels of the *Kr-h1* transcription factor are positively correlated with ovarian activation in worker bees [33]. Genome-wide hypomethylation induced by the demethylating agent Decitabine (C_8_H_12_N_4_O_4_) can disrupt reproductive suppression, triggering aggressive behavior and ovary development in worker bees [5,34], further underscoring the pivotal role of DNA methylation in the regulation of honeybee social behavior.

In bees, caste differentiation exemplifies how individual developmental fate is jointly regulated by nutritional signals and epigenetic mechanisms. Among these mechanisms, DNA methylation functions as a central regulatory factor, playing pivotal roles in tissue-specific transcription, alternative splicing, and cell fate determination. The effects of DNA methylation show a clear dependence on genome structure. Genes with low CpG density are typically involved in basic metabolic processes, where promoter methylation is strongly negatively correlated with transcriptional activity. In contrast, genes with high CpG density are primarily associated with neurodevelopment and the regulation of social behavior, and methylation within their exonic regions modulates alternative splicing, thereby promoting functional diversity [10,35]. As a eusocial insect, *Apis mellifera* has evolved an epigenetic landscape adapted to its social organization. Multiple studies have demonstrated significant differences in DNA methylation profiles across castes—queens, workers, and drones—and tissues such as the brain and ovaries, suggesting caste-specific epigenetic regulation. For instance, methylation levels in the fat body are notably higher than those in the brain [12]. During the larval stage of worker bees, genome-wide methylation levels drop by approximately 25%, but increase again in the adult head, potentially reflecting behavioral transitions and social role specialization [36]. In reproductive cells, oocytes exhibit significantly higher methylation levels than sperm [37]. In early embryogenesis, the median level of CG methylation reaches as high as 83% [36], which stands in stark contrast to other insects such as *Anopheles mosquitoes*, where CG methylation is generally low during embryonic stages [38], suggesting that epigenetic reprogramming may play a role in germ cell fate determination.

Cross-species comparative studies across honeybee lineages have further revealed significant divergence in non-CG methylation, particularly at CHH sites. For instance, European honeybees (EHB) exhibit markedly higher intronic CHH methylation levels than Africanized honeybees (AHB). This differential methylation is proposed to influence alternative splicing via selective recruitment of splicing factors mediated by 5-hydroxymethylcytosine (5-hmC), leading to the generation of adaptive isoforms [39]. In the context of body color regulation, thousands of differentially methylated sites (DMSs) have been identified in hybrid queens, particularly within KEGG pathways such as tyrosine metabolism. Some of the differentially methylated genes (DMGs) show direct associations with melanin biosynthesis, reinforcing the involvement of DNA methylation in phenotypic shaping [40]. Developmental divergence between queens and workers is reflected at the molecular level through distinct methylation trajectories. Queen bee larvae have a genome-wide methylation level approximately 30% lower than worker bees during critical developmental windows, such as the peak period of pharyngeal lateral organ activity [41]. However, during the prepupal and pupal stages, methylation levels in queens increase sharply, reaching 15% and 21%, respectively, in stark contrast to 2.5–4% in workers [42]. These DMGs are enriched in pathways related to the cytoskeleton, signal transduction, and neurodevelopment, with region-specific expression patterns observed in the larval brain [43,44]. Functional perturbation studies further confirm the role of DNA methylation in developmental fate determination. Suppressing *Dnmt3* expression, via RNA interference (RNAi) or decitabine treatment, relieves the repression of worker-specific genes (e.g., cuticular proteins), promoting a queen-like developmental trajectory [45,46,47]. Consistently, DNA methylation levels in queen-destined larvae are lower than those in worker-destined individuals [48]. In individuals experiencing developmental perturbations, methylation levels of genes such as *dynactin* significantly decrease, approaching levels observed in queen bees. This demonstrates the reversibility of DNA methylation in regulating developmental programs [20,49].

#### 2.2.2. Evolutionary Conservation

Although DNA methylation exhibits high tissue specificity, its methylation pattern remains remarkably stable across generations and is more readily inherited in bees than in mammals [50]. In Yagound’s study, samples derived from the same patriline shared twice as many methylated sites and only one-quarter as many differentially methylated regions compared to samples from different patrilines. Notably, no evidence of DNA methylation reprogramming was observed in honeybees. Research has demonstrated that approximately 81% of methylation sites are completely conserved between offspring and parental individuals [50,51,52], indicating that epigenetic marks can be stably maintained across generations, although the influence is generally less pronounced than that of generational genetic effects [52]. This stability nonetheless remains plastic under specific ecological or artificial interventions. For example, developmental stress induced by commercial queen breeding systems can lead to the accumulation of methylation variation, which can be transmitted to subsequent generations [53]. Especially in male germ cells, DNA methylation exhibits co-variation with genetic variants such as SNPs, constructing a dual-layer regulatory system interwoven with both genetic and epigenetic factors, providing a basis for caste fate diversity [54]. Contrastingly, Cardoso reported a contrasting perspective: although thousands of differentially methylated regions were identified, no significant association between DNA methylation and gene expression was found in the examined genes [55]. Only three genes (GB42836, GB49839, and GB54664) showed differential expression in specific tissues. Interestingly, the presence or absence of the queen affected the expression of all four Dnmt genes. Cardoso concluded that DNA methylation is not a primary driver of gene expression reprogramming in the brains and ovaries of young worker bees [55].

## 3. Non-Coding RNAs

In 1998, Craig C. Mello and Andrew Z. Fire’s team first uncovered the phenomenon of double-stranded RNA-mediated gene silencing using a *Caenorhabditis elegans* model, thereby formally proposing the mechanism of RNA interference (RNAi) [56]. This landmark discovery earned the 2006 Noble Prize in Physiology or Medicine. This groundbreaking discovery not only revolutionized the theoretical framework of gene expression regulation but also laid a critical foundation for subsequent epigenetic research in model organisms such as the honeybee. Recent studies have shown that various types of non-coding RNAs finely modulate gene expression at both transcriptional and translational levels, influencing physiological processes such as queen–worker differentiation, learning and memory, and responses to environmental stress [57]. Among these, long non-coding RNAs (lncRNAs), microRNAs (miRNAs), and small interfering RNAs (siRNAs) constitute key components of epigenetic regulation in honeybees.

**Figure 2:**
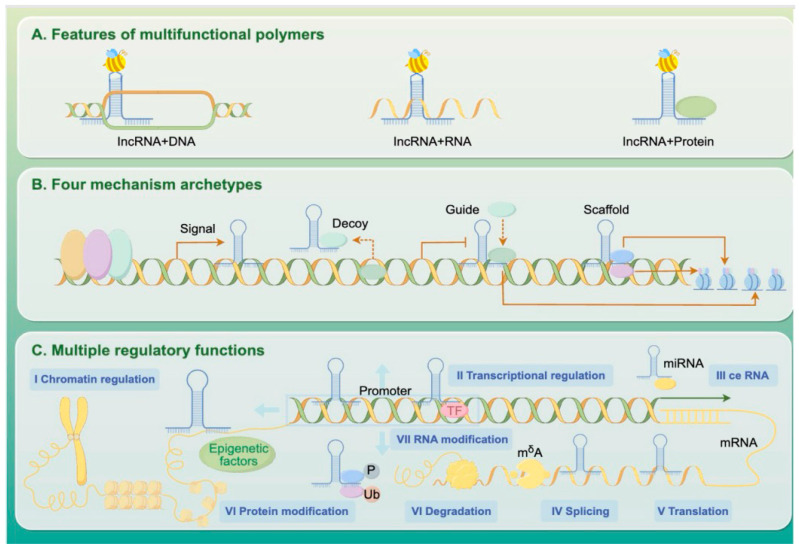
Mechanisms of lncRNA-Mediated Gene Expression Regulation.

Long non-coding RNAs (lncRNAs) are transcripts longer than 200 nucleotides that do not encode proteins but play essential roles in gene regulation. These lncRNAs can bind to DNA, recruit chromatin-modifying complexes to reshape epigenetic landscapes, interact with mRNAs to influence their splicing or stability, and serve as scaffolds or decoys for regulatory proteins (Figure 2).

Long non-coding RNAs (lncRNAs), as key molecular regulators in the epigenetic landscape of honeybees, coordinate physiological development and social behavioral plasticity through multilayered mechanisms. Lncov1 and Lncov2 are the first lncRNAs identified in honeybees to be associated with reproductive division of labor in eusocial insects. These lncRNAs play crucial roles in regulating ovarian development and caste differentiation, marking a seminal discovery to the study of bee lncRNAs [58]. The lncRNA Nb-1 participates in sex differentiation and caste determination through various mechanisms. In haploid male embryos, Nb-1 modulates male-specific developmental programs by delaying the zygotic genome activation (ZGA) process. In contrast, in diploid female embryos, 73% of intron-derived lncRNAs are co-expressed with the three major ZGA peaks [59]. At the level of adult behavior, the transition from nurse bees to foragers is accompanied by a dynamic reprogramming of head lncRNA expression profiles. The Wnt signaling pathway drives adaptive changes in olfactory recognition by regulating alternative splicing of olfactory receptor genes [60]. Notably, Nb-1 exhibits multifunctionality in worker bee brains. Its high expression in young workers promotes behavioral transitions by activating octopaminergic neurons [61], whereas its specific enrichment in mushroom body neural stem cells during the pupal stage contributes to the construction of neural circuits involved in learning and memory [62]. This mechanism may provide a molecular basis for the complex social behavior of the waggle dance in bees: nitrogen metabolism-related lncRNAs(including those co-expressed with genes involved in amino acid biosynthesis (e.g., glutamine synthetase [GS], glutamate dehydrogenase [GDH]) modulate neurotransmitter synthesis pathways, thereby affecting the signal precision of dance movements [63]. Evolutionary analyses indicate that while Nb-1 is conserved within the Apidae family, it has undergone functional innovation to simultaneously regulate oocyte maturation and adult neural plasticity—illustrating the dynamic balance maintained by lncRNAs between developmental robustness and ecological adaptability.

Long non-coding RNAs (lncRNAs) function as central hubs in multidimensional epigenetic regulatory networks that enable honeybees to respond to external stressors and maintain physiological homeostasis. Under pesticide exposure scenarios, lncRNAs regulate adaptive neural functions through dynamic chromatin remodeling. Under thiamethoxam stress, the long lncRNA LOC102654625 exerts regulatory roles in orchestrating toxic responses [64]. Similarly, imidacloprid exposure induces reprogramming of brain lncRNA expression, suppressing histone acetylation of genes involved in mitochondrial electron transport chain function, thereby synchronously regulating compensatory energy metabolism and activation of immune responses [65]. In temperature stress studies, lncRNAs modulate the balance of transcriptional isoforms of heat shock protein (HSP) genes through alternative splicing. RNAi-mediated silencing of these lncRNAs leads to dysregulation of HSP70/90 expression, disrupts coordination between heat shock factors (HSFs) and DNA damage repair pathways, and ultimately triggers apoptotic cascades [66]. In response to predator attacks, the lncRNA kakusei, expressed in type II Kenyon cells, regulates heat production and thermosensation during thermal defense behaviors, facilitating the formation of heat-ball clusters to resist hornet attacks [67]. In the context of pathogen defense, lncRNAs demonstrate multimodal immune regulatory roles. For instance, during chalkbrood infection, larval gut-specific lncRNAs cis-regulate neighboring genes in the Jak-STAT pathway through H3K27me3 histone modification, while simultaneously functioning as competing endogenous RNAs (ceRNAs) by sponging ame-miR-2765, thus exerting dual control over phagophore formation and antimicrobial peptide expression [68,69]. Upon infection with *Nosema ceranae*, lncRNAs build dynamic interaction networks (e.g., lncRNA_1024 → ame-miR-34 → Toll receptor) to precisely regulate humoral immunity and oxidative stress balance, with regulatory strength being dose-dependent on spore load [70,71]. The latest mechanistic studies reveal that the brain-specific lncRNA LOC113219358 forms a nucleocytoplasmic shuttling complex with MAPK phosphatases, coordinating neuroinflammatory responses while maintaining synaptic plasticity in the mushroom body—thus enabling cross-system integration of immune and neural functions [72]. Evolutionary genomics analyses further show that among 11 parasite-responsive lncRNAs, 10 exhibit sequence conservation across Hymenopteran insects. However, their regulatory targets have undergone honeybee-specific expansion toward genes associated with social behavior, suggesting that natural selection has driven functional innovation in non-coding regions to support socialized immune adaptations in bees [73].

**Figure 3:**
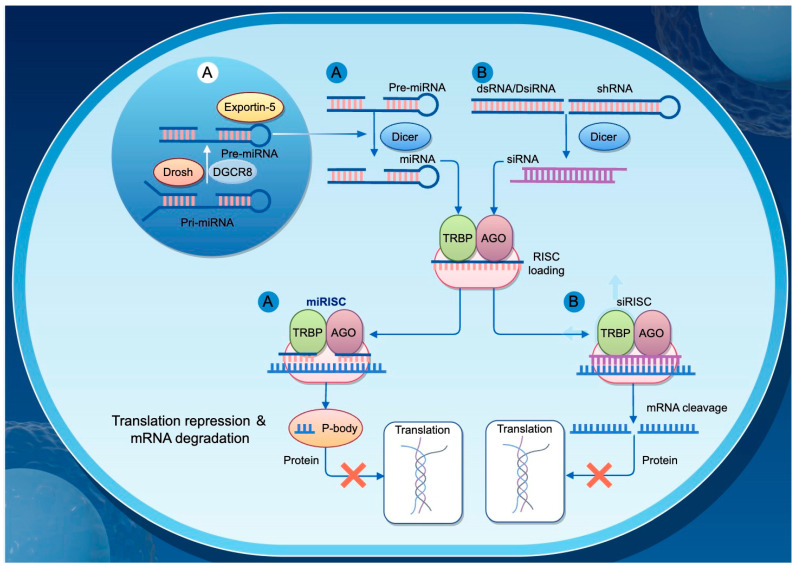
Mechanisms of Gene Regulation by siRNA and miRNA. This diagram illustrates the distinct pathways through which small interfering RNAs (siRNAs) and microRNAs (miRNAs) regulate gene expression in eukaryotic cells. (A) miRNA biogenesis begins in the nucleus, where primary miRNAs (pri-miRNAs) are processed by Drosha and its cofactor DGCR8 into precursor miRNAs (pre-miRNAs). These pre-miRNAs are then exported to the cytoplasm via Exportin-5. In the cytoplasm, Dicer processes the pre-miRNAs into mature miRNAs, which are loaded into the RNA-induced silencing complex (RISC), consisting of TRBP and AGO proteins, forming the miRISC. The miRISC typically binds to partially complementary sequences in target mRNAs, leading to translational repression and mRNA degradation. (B) siRNAs are derived from double-stranded RNA (dsRNA), Dicer-substrate siRNA (DsRNA), or short hairpin RNA (shRNA), and are processed by Dicer into short double-stranded siRNAs. These siRNAs are then incorporated into the RISC to form si-RISC, which generally binds perfectly complementary target mRNAs and induces their cleavage, thereby inhibiting protein expression.

MicroRNAs (miRNAs) are a class of single-stranded non-coding RNA molecules approximately 22 nucleotides in length and are widely present across eukaryotic organisms. They regulate gene expression by binding partially complementary sequences in the 3′ untranslated region (3′-UTR) of their target mRNAs, thereby inhibiting translation or promoting mRNA degradation. miRNAs play essential roles in numerous biological processes, including cell differentiation, development, metabolism, and immune responses (Figure 3).

In honeybees, one of the most highly eusocial insects, microRNAs (miRNAs) serve as key regulators within dynamic, multilayered post-transcriptional regulatory networks, orchestrating individual behavioral differentiation, colony-level coordination, and environmental adaptability. Specific miRNAs play pivotal roles in the temporal regulation of labor division. For example, the honeybee-specific ame-miR-2796, which has co-evolved with its host gene PLC-epsilon, targets neural differentiation pathways to facilitate the behavioral transition from nurse bees to foragers. Conversely, exogenous supplementation of miR-184 in royal jelly can induce queen-destined adult bees to display worker-like phenotypes [74]. Additionally, ame-miR-279a downregulates the expression of the transcription factor Mblk-1, lowering the sugar response threshold and promoting the initiation of foraging behavior [75]. In the context of social information processing, the miR-34 family enhances pheromone recognition via Notch pathway modulation of olfactory receptor activity. miR-124 and ame-miR-278/282 contribute to the neural basis of complex behaviors such as the waggle dance by regulating synaptic plasticity and neurometabolic pathways, respectively [76,77]. miRNAs also establish type-specific and functionally modular regulatory networks in response to environmental stress. Pesticide exposure induces widespread miRNA expression changes. For example, ame-miR-3786-3p targets CDK5 to regulate behavioral transition thresholds, while ame-miR-3049-5p enhances antimicrobial peptide synthesis by inhibiting Tollip, forming an immune-behavioral co-defensive mechanism [78]. Exposure to thiamethoxam specifically activates ame-miR-6038, which disrupts stress granule assembly by binding the 3′-UTR of HSP90, inducing abnormal splicing of genes such as synaptotagmin-7, and ultimately reducing homing ability by 41% in worker bees [79]. Under pathogenic stress, ame-miR-317 inhibits mitochondrial pyruvate carrier MPC1 expression [80], shifting metabolic pathways toward glycolysis (the Warburg effect), thus providing a metabolic basis for parasite proliferation [81]. Furthermore, miR-13a and members of the let-7 family target PGRP-LB and MKP-1 to activate the Toll signaling pathway, initiating a rapid immune response [82]. Evolutionary genomics reveals that 24–72% of bee-specific miRNAs (e.g., miR-305) lack known homologs yet are consistently found across 12 eusocial bee species [83]. Targeted knockdown of abdominal ame-miR-305-5p induces transcriptional changes in the brain, notably impacting genes encoding transcription factors known to regulate behavioral maturation [84].

At the level of developmental fate determination, microRNAs (miRNAs) regulate caste differentiation, ovarian activation, and transgenerational phenotypic shaping. Systematic differences in miRNA expression are observed between haploid males and diploid females. For instance, miR-276b modulates the expression of *Dnmt3*, affecting juvenile hormone (JH) synthesis and promoting ovarian development in queens, while drone worker differentially expressed miRNAs regulate hypopharyngeal gland development via the PI3K/Akt/mTOR signaling pathway [76]. In queen larvae, elevated expression of ame-let-7—at twice the level found in worker larvae—suppresses the Notch signaling pathway and promotes ovariole primordium development. Conversely, in worker larvae, high expression of ame-miR-263 targets *FoxO*, accelerating JH degradation and committing individuals to the worker developmental trajectory [85]. Semen is enriched with miR-34 and miR-210, which regulate the alternative splicing of neurodevelopmental genes such as *elav* and *brat*, enhancing phototactic responses in the offspring [86,87]. Queen pheromones suppress ovarian activation in workers by modulating miR-100, which in turn inhibits vitellogenin synthesis [88]. Moreover, miRNAs serve as cross-species information vectors linking nutritional signals to ecological inputs. Several plant-derived miRNAs can regulate honeybee gene expression via the food chain, significantly repressing *amTOR* expression, delaying chitin deposition, and impacting developmental timing and gut stem cell proliferation [89,90]. Overall, miRNAs establish an ecologically adaptive epigenetic regulatory network through a ternary strategy: the reprogramming of conserved pathways, construction of novel gene networks, and stress recognition and memory encoding—thus contributing to individual behavior, colony-level health, and transgenerational inheritance in honeybees (Table 1).

Small interfering RNAs (siRNAs) are double-stranded RNA molecules approximately 21–23 nucleotides in length, typically generated from exogenous double-stranded RNA via cleavage by the Dicer enzyme. siRNAs guide the RNA-induced silencing complex (RISC) to perfectly base pair with their target mRNAs, leading to the degradation of the mRNA and the silencing of specific gene expression. Integrated bioinformatic analyses have established a regulatory network composed of lncRNAs, miRNAs, and mRNAs, revealing their dynamic and coordinated interactions in honeybee epigenetic regulation. These intricate molecular interactions potentially coordinate gene expression in bees by modulating various signaling pathways, including Hippo, Wnt, and TGF-beta pathways [91] (Figure 3).

RNA interference (RNAi) is a conserved gene-silencing mechanism that has been widely used to suppress target gene expression in honeybees and other insects. RNA interference (RNAi) technology has emerged as a crucial tool for gene function studies in honeybees, demonstrating regulatory potential across multiple biological levels. In behavioral development, silencing of the *Vitellogenin* gene resulted in a 67% reduction in its expression, leading to decreased protein levels in the hemolymph, accompanied by increased juvenile hormone (JH) levels and upregulation of its receptor *usp*, revealing an endocrine role in the behavioral transition of worker bees [92]. In a study of immune mechanisms, engineered gut bacterium delivers targeted RNAi through sustained dsRNA production, effectively suppressing viral infections and controlling mite parasitism in honey bee [93]. Neuroethological research has revealed that silencing *MRJP1* significantly reduced the proboscis extension response rate of honeybees during the third to fifth rounds of training, highlighting its role in learning ability [94]. At the developmental level, RNAi targeting the IRS/TOR pathway elevated DNA methylation levels in larval fat bodies, which redirected development from queen to worker phenotype, suggesting that this signaling pathway regulates caste differentiation through epigenetic mechanisms [95]. Furthermore, the Li-Byarlay group developed a spray-based RNAi technique that reduced *DNMT3* expression in tracheal cells by 30%, triggering an increase in exon skipping and a decrease in intron retention. This subsequently altered the isoform ratios of genes related to labor division, revealing that DNA methylation influences behavioral plasticity by regulating the chromatin-binding capacity of splicing factors such as *SRSF5* [96]. Collectively, RNAi serves not only as a means for functional gene validation but also provides an efficient intervention strategy for elucidating the behavioral, physiological, and epigenetic regulatory mechanisms in honeybees.

RNA interference (RNAi), through its dual mechanism of targeted gene silencing and nonspecific antiviral activation, has become one of the core strategies for environmentally friendly control of honeybee pathogens and parasites. On one hand, RNAi enables efficient silencing of specific viral genes. Studies have shown that oral administration of double-stranded RNA (dsRNA) to honeybees targeting Israeli acute paralysis virus (IAPV) significantly reduces viral loads, and IAPV-specific small interfering RNAs (siRNAs) can be detected in honeybee tissues, indicating that the dsRNA is recognized by Dicer and triggers the RNAi pathway [97]. This strategy also exhibits good stability under practical beekeeping conditions [98]. Desai et al. further demonstrated that feeding dsRNA specific to deformed wing virus (DWV) significantly reduces infection levels and mortality, while alleviating wing deformity symptoms in bees [99]. On the other hand, RNAi can also induce broad-spectrum antiviral responses in honeybees. Using Sindbis virus (SINV-GFP) as a model, Flenniken and colleagues found that not only SINV-specific dsRNA but also non-specific dsRNAs—such as poly(I:C), LUC dsRNA, and DCV dsRNA—significantly reduced viral infection levels [100,101], suggesting that RNAi may activate a sequence-independent innate immune mechanism. Beyond viruses, RNAi also shows promise in parasite control. Paldi et al. designed dsRNA targeting the ADP/ATP translocase of *Nosema ceranae*, and feeding it to honeybees significantly decreased infection levels and reduced spore counts by approximately two-thirds. Additionally, bees showed lower sucrose response thresholds [102], indicating that RNAi may help mitigate the metabolic damage inflicted by parasitic infection. In the control of *Varroa destructor*, targeting several key genes in the mite—such as RNA polymerase III, vacuolar H+-ATPase, and inhibitors of apoptosis (*iap1/2*)—using dsRNA reduced their expression levels by 35% to 60%, significantly decreasing mite loads in bee colonies by an average of 53% to 61% [103]. This study also provided the first evidence that dsRNA ingested by honeybees can be transferred trophically to parasitic mites, highlighting RNAi’s unique mechanism in cooperative control of parasitic organisms.

## 4. Histone Modifications

Histones are basic proteins present in eukaryotic somatic cell chromatin. Each core histone comprises two distinct structural domains: a globular folded domain and an amino-terminal (N-terminal) domain. The globular folded domain is involved in interactions between histones and contributes to the wrapping of DNA into a helical structure, whereas the N-terminal domain extends outside the globular core of the nucleosome, serving as signaling sites frequently modified covalently by histone acetyltransferases, histone methyltransferases, and histone kinases [104].

Histone modifications, including acetylation and methylation, can dynamically alter chromatin conformation in honeybees, thereby affecting gene transcriptional activity. Dickman et al. systematically analyzed modification profiles of histones H3 and H4 using mass spectrometry, extracting histones from 96-h-old larvae and queen ovaries of honeybees. They identified 23 specific modification states involving 23 distinct peptide segments. These modifications included lysine acetylation (ac) and methylation (me), exhibiting tissue-specific patterns [105].

Histone modifications play critical roles in environmental responses and caste differentiation in honeybees. For instance, heat stress increases methylation of H3K4m2 and H3K4m3, while enhancing demethylation of H3K27m2 and H3K27m3 at the l(2)elf gene, demonstrating that methylation/demethylation of histone H3K4 and H3K27 represents a key epigenetic mechanism regulating gene expression under heat stress in honeybees [106]. Treatment with 0.1 mM spermidine results in reduced acetylation of histone H3 at lysine residues K18 and K27, increased acetylation at K9, and no alteration at K14, collectively contributing to anti-aging effects in honeybees [107]. The H3K4me1 modification is considered a crucial factor for establishing and maintaining caste-specific transcriptional programs, whereas modifications such as H3K27ac and H3K4me1 influence queen–worker differentiation [108]. Phenotypic polymorphisms in honeybees, such as queen and worker castes, are predominantly driven by nutritional differences. Specifically, 10-HDA from royal jelly inhibits histone deacetylase (HDAC) activity, with a half-maximal inhibitory concentration (IC50) ranging between 5 and 8 mM, thereby modulating histone acetylation levels and subsequent gene expression [4,109,110,111]. Notably, caste differentiation between queens and workers depends not only on DNA methylation differences mediated by enzymes like DNMT but also closely involves the histone modification H3K4me1 [112].

Histone modifications and DNA methylation are tightly interconnected epigenetic mechanisms that coordinate gene regulation. Hunt et al. analyzed gene expression data from fire ants, honeybees, and *Drosophila* to evaluate the associations among DNA methylation, histone modifications, and gene expression. They found that H3K4me2/me3 marks are closely associated with transcription initiation and are primarily localized to the 5′ regions of genes. H3K79me2 and H3K36me3 are associated with transcriptional elongation and are mainly located within exonic regions. These modifications show a significant positive correlation with DNA methylation levels. In contrast, H3K27me2/me3, which are linked to polycomb-mediated transcriptional repression, and H3K9me2/me3, which are associated with heterochromatin formation and gene silencing, exhibit a negative correlation with DNA methylation levels [113]. These findings confirm that DNA methylation and histone modifications collaboratively regulate transcriptional activity in honeybees, as summarized in Table 2, reflecting their synergistic epigenetic interplay [113]. DNA methylation may influence the binding of transcription factors or the accessibility of chromatin, thereby affecting the distribution and function of histone modifications. This cooperative mechanism enables precise regulation of gene expression in response to various physiological and environmental conditions in honeybees.

**Table 2:** Bee histone modifications.

Histone Site	Modification Type	Modification Pattern	Function	Reference
H3K4	me1, me2	Unmodified form is the most abundant; me1 level is low; me2 is undetectable	Associated with gene promoters, typically linked to gene activation; me1 influences caste differentiation	[105,106,108,114]
H3K9	ac, me1, me2, me3	me1 is the most abundant; me2 and me3 levels are low	me1 is associated with enhancers; me2 and me3 are linked to gene repression	[105]
H3K14	ac	High acetylation level	Associated with gene promoters, typically linked to gene activation	[105]
H3K18, H3K23	ac	Monoacetylation of H3K23 is more abundant than deacetylation	Associated with gene promoters, typically linked to gene activation	[105]
H3K27	me1, me2, me3	me2 is the most abundant	me2 is linked to gene transcription; me3 is associated with gene silencing	[105,106,108]
H3K36	me1, me2, me3	Low modification levels	me1 is associated with gene transcription regions	[105]
H3K79	me1, me2	level me1 is higher; me2 is lower	Associated with gene transcription regions, typically linked to gene activation	[105]
H4K5, H4K8, H4K12, H4K16	ac	H4K16 acetylation is the highest, followed by H4K8, H4K12, and H4K5	Typically linked to gene activation	[105]
H4K20	me1, me2	level me1 is higher; me2 is lower	Associated with chromosome structure and stability, also linked to gene activation	[105]

## 5. Discussion

Research on DNA methylation in honeybees has revealed its central role in caste differentiation and behavioral plasticity. Notably, recent attention to RNA methylation modifications provides novel insights into epigenetics. Studies indicate that transcripts with higher levels of m6A methylation generally exhibit lower expression, possibly mediated by recruitment of specific RNA-binding proteins (YTHDF2), which recognize and bind m6A-modified RNA to promote its degradation [115]. Additionally, m6A RNA modifications impact larval development and caste determination in honeybees, with global m6A levels varying in worker fat bodies and brain tissues [116]. However, the molecular mechanisms by which DNA or RNA methylation mediates specific gene expression changes affecting bee behavior remain unclear. Furthermore, the reversibility of DNA methylation and its dynamic regulation throughout individual development and behavioral shifts require further exploration. For instance, systematic experimental evidence is still lacking regarding how methylation patterns of specific genes are remodeled during different behavioral stages and whether these changes are heritable.

Non-coding RNAs (ncRNAs), such as microRNAs (miRNAs) and small interfering RNAs (siRNAs), have increasingly garnered attention in research exploring behavioral regulation, immune responses, and environmental adaptability in honeybees. miRNAs modulate gene expression by inhibiting mRNA, thereby influencing neural development, metabolism, and immunity-related genes, which in turn affect learning, memory, social behavior, and stress resistance in honeybees. siRNAs primarily function within the RNA interference (RNAi) mechanism, demonstrating therapeutic potential for controlling viral infections and parasite infestations in honeybees. Previous studies have investigated RNAi-based strategies for honeybee virus management; however, research on ncRNAs remains limited. For example, despite identifying numerous miRNAs linked to honeybee behavior and immunity, the precise identification of their target genes continues to present challenges. Furthermore, current studies on environmental factors—such as pesticide exposure and pathogen infections—affecting miRNA expression in honeybees are predominantly laboratory-based. It remains unclear whether such stressors impact the long-term health of bee colonies under natural conditions, highlighting the need for further field-based research. Future research would benefit from longitudinal miRNA profiling in apiaries, which could reveal how small RNAs dynamically respond to environmental factors such as seasonal changes, pathogen exposure, or pesticide accumulation. Such field-based approaches may help bridge the gap between controlled laboratory studies and natural colony-level epigenetic regulation.

Histone modifications represent another critical epigenetic regulatory mechanism, significantly influencing gene expression and behavioral plasticity in honeybees. Studies indicate that methylation of histone H3K4 correlates positively with gene activation, whereas demethylation of histone H3K27 facilitates transcription of specific genes, suggesting essential roles for these modifications in environmental stress responses and behavioral decisions in honeybees. Notably, under heat stress conditions, elevated methylation of H3K4me2 and H3K4me3, accompanied by increased demethylation of H3K27me2 and H3K27me3, underscores histone modifications as pivotal regulatory mechanisms enabling bees to adapt to extreme temperatures. Furthermore, 10-HDA, a constituent of royal jelly, inhibits histone deacetylase (HDAC) activity, thereby influencing gene expression patterns and promoting queen development. Despite these advances highlighting associations between histone modifications and honeybee phenotypic diversity, the detailed molecular mechanisms underlying gene regulation remain elusive. Future research should clarify potential synergistic interactions among different histone modifications and explore how these modifications collaborate with DNA methylation and non-coding RNAs to jointly regulate honeybee behavior.

Recent studies have shown that the complex social phenotypes of honeybees are shaped by the synergistic interplay of DNA methylation, non-coding RNAs, and histone modifications. For instance, in investigations of worker behavioral plasticity, DNA methylation suppresses the promoter activity of synaptic plasticity-related genes such as *neurexin I*, thereby reducing their expression. Meanwhile, brain-specific lncRNAs (e.g., LOC113219358) recruit histone deacetylases (HDACs) to further condense chromatin structure, forming a dual-layer silencing mechanism [28,72]. This “methylation–lncRNA–histone modification” multilayered epigenetic regulation may provide a molecular basis for the rapid environmental responsiveness observed in honeybees. In addition, miRNAs such as ame-miR-34 indirectly modulate genome-wide methylation patterns by targeting *Dnmt3*, thereby influencing caste differentiation [76]. However, current research still faces several technical challenges: limited single-cell resolution hampers the analysis of epigenetic heterogeneity; the mechanisms underlying transgenerational inheritance remain unclear, restricting our understanding of environmental epigenetic memory; and delayed integration of multi-omics data impedes the construction of comprehensive regulatory networks. Future studies should integrate single-cell epigenomics (e.g., scATAC-seq), CRISPR-dCas9-based targeted epigenome editing, and deep-learning-based multi-omics integration frameworks (e.g., MOFA+, DeepMOCCA), to elucidate the cell-type specificity, transgenerational inheritance, and phenotypic impacts of epigenetic modifications in bees.

Despite significant progress in honeybee epigenetics, core controversies remain unresolved. First, the functional conservation of DNA methylation is uncertain. Unlike the classical mammalian model where promoter methylation directly represses gene expression, honeybee-specific exon methylation may indirectly influence phenotype by regulating mRNA splicing events such as intron retention or exon skipping [32]. This discovery challenges the traditional notion of “universal methylation functionality”, suggesting that social insects might have evolved unique epigenetic regulatory strategies. Second, the mechanisms by which environmental signals are converted into epigenetic modifications remain elusive. For instance, although royal jelly-derived 10-HDA inhibits histone deacetylase (HDAC) activity [110], the pathway involving its intestinal absorption, transport via hemolymph, and eventual chromatin remodeling in reproductive or neuronal cells remains unclear. Utilizing honeybee-organoid models in combination with synthetic epigenetic perturbation systems (e.g., optically controlled HDAC inhibitors) may offer breakthroughs in elucidating this “nutrition-epigenetics” signaling pathway.

Despite ongoing controversies, the applied potential of honeybee epigenetics is becoming increasingly evident. Techniques leveraging RNA interference (RNAi) for targeted epigenetic regulation have shown practical effectiveness; for instance, feeding bees with dsRNA targeting the inhibitor of *apoptosis* gene in *Varroa destructor* mites reduced infestation rates by 53–61% [103], providing a novel paradigm for eco-friendly pest control. Additionally, the expression changes in pesticide-responsive lncRNAs (e.g., LOC102654625) appear to reflect colony health risks earlier than conventional mortality indicators [65], highlighting their promise as new biomarkers for environmental monitoring. Moving forward, integrating insights from epigenetic mechanisms with practical colony management approaches—such as disease-resistant breeding and precision feeding—may accelerate the development of innovative strategies to combat pollinator decline. Such advancements ultimately contribute to global ecological stability and agricultural sustainability, in alignment with the United Nations Sustainable Development Goals, particularly Goal 2 (Zero Hunger) and Goal 15 (Life on Land).

## 6. Conclusions

Epigenetic mechanisms orchestrate honeybee development, behavior, and adaptation, yet key questions remain. However, critical controversies persist, including uncertainties regarding the functional conservation of DNA methylation and the specific molecular pathways linking environmental signals to epigenetic changes. Addressing these unresolved issues calls for interdisciplinary strategies, including CRISPR-epigenome editing, single-cell profiling, and integrative multi-omics, to dissect how epigenetic modifications drive phenotypic plasticity in honeybees. Additionally, the emerging applications of epigenetic techniques, such as RNA interference for pest and pathogen management and non-coding RNA biomarkers for environmental monitoring, highlight practical opportunities to enhance bee health management and contribute positively to global agricultural sustainability and ecological security. Such knowledge can help mitigate pollinator declines caused by pests and pathogens globally.

## Figures and Tables

**Table 1:** Functionally Classified miRNAs in *Apis mellifera*.

miRNA	Target Gene/Pathway	Function/Effect	Category	Reference
ame-miR-3786-3p	*CDK5* (regulates behavioral transition thresholds)	Modulates foraging behavior under pesticide stress	Pesticide Response	[78]
ame-miR-3049-5p	*Tollip* (enhances antimicrobial peptide synthesis)	Activates immune response under stress conditions	Pesticide Response	[78]
ame-miR-6038	*HSP90* (disrupts stress granule assembly, affects synaptotagmin-7)	Reduces homing ability by 41% after thiamethoxam exposure	Pesticide Response	[79]
ame-miR-317	*MPC1* (suppresses mitochondrial transport, shifts metabolism to glycolysis)	Supports parasite proliferation through metabolic reprogramming	Pesticide Response	[80,81]
Plant-derived miRNAs	*amTOR* (regulates development, chitin deposition, and gut stem cell proliferation)	Delays development and chitin formation; impacts gut regeneration through nutritional input	Pesticide Response	[89,90]
ame-miR-2796	*PLC-epsilon* (neural differentiation)	Facilitates nurse-to-forager transition (behavioral maturation)	Behavioral Regulation	[74]
miR-184	Unspecified (affects caste trajectory)	Induces worker-like traits in queen-destined bees (via royal jelly)	Developmental Plasticity	[74]
ame-miR-279a	Mblk-1 (transcription factor)	Lowers sugar response threshold; promotes foraging behavior	Behavioral Regulation	[75]
miR-34 family	Notch signaling (olfactory modulation)	Enhances pheromone recognition via olfactory receptor activity	Behavioral Regulation	[76,77,86,87]
miR-124	Neural synaptic regulators	Regulates synaptic plasticity related to complex behaviors (e.g., waggle dance)	Behavioral Regulation	[76,77]
ame-miR-278/282	Neurometabolic pathways	Modulates brain metabolic states during behavior transitions	Behavioral Regulation	[76,77]
ame-miR-305	Transcription factors for behavioral maturation	Alters gene expression in brain; regulates caste-associated behaviors	Evolutionary/Behavioral	[84]
miR-276b	*Dnmt3* (DNA methyltransferase)	Modulates JH synthesis; promotes queen-like ovarian development	Developmental Regulation	[76]
ame-miR-263	*FoxO* (transcription factor)	Accelerates juvenile hormone degradation; biases toward worker fate	Developmental Regulation	[85]
ame-let-7	*Notch* (suppression)	Promotes ovariole primordium development in queen larvae	Developmental Regulation	[85]

## Abbreviations

The following abbreviations are used in this manuscript:

AEB	Alternative exon boundaries
AC	Acetylation
ATE	Alternative terminal exons
CH3	Methyl group
CSBV	China Sac brood virus
CpG	Cytosine–phosphate–guanine
DWV	Deformed wing virus
DMRs	Differentially methylated regions
DNA	Deoxyribonucleic acid
DNMT	DNA methyltransferase
DsRNA	Double strand RNA
ES	Exon skipping
GFP	Green fluorescence protein
H3	Histone H3
H3K27me2	Histone 3 lysine 27 demethylation
H3K27me3	Histone 3 lysine 27 trimethylation
H3K36	Histone 3 lysine 36
H3K36me3	Histone 3 lysine 36 trimethylation
H3K4	Histone 3 lysine 4
H3K4me2	Demethylation of histone H3 lysine 4
H3K4me3	Trimethylation of histone H3 lysine 4
H3K79	Histone 3 lysine 79
H3K79me2	Histone 3 lysine 79 demethylation
HATs	Histone acetyltransferases
HDACs	Histone deacetylases
HSF	Heat shock transcription factor
HSP	Heat shock proteins
IAPV	Israeli acute paralysis virus
IR	intron retention
KEGG	Kyoto Encyclopedia of Genes and Genomes
lncRNA	Long non-coding RNA
Me	Methylation
miRNAs	MicroRNAs
mRNA	Messenger RNA
ncRNA	Non-coding RNA
Poly I:C	Polyinosinic acid-polycytidylic acid
RISC	RNA-Induced silencing complex
RNA	Ribonucleic acid
RNAi	Ribonucleic acid interference
SAM	S-adenosylmethionine
siRNA	Small interference RNA
TCA	Tricarboxylic acid cycle
TOR	Phosphatidylinositol/rapamycin target protein
Zeb	Zebularine
ZGA	Zygotic genome activation
